# Isolation and genetic analysis of mycobacteria from suspect tuberculous lesions in slaughtered cattle from Wolaita, Ethiopia

**DOI:** 10.1099/acmi.0.000915.v3

**Published:** 2025-03-18

**Authors:** Melese Yilma Zaba, Sebsib Neway, Damien Farrell, Eva Denion, Viktor Perets, Melaku Tilahun, Kidist Bobosha, Joseph P. Cassidy, Asefa Asmare, Stephen V. Gordon

**Affiliations:** 1 School of Veterinary Medicine, Wolaita Sodo University, Wolaita, Ethiopia; 2 School of Veterinary Medicine, University College Dublin, Belfield, Dublin, Ireland; 3 Armauer Hansen Research Institute, Addis Ababa, Ethiopia; 4 Polytech Clermont, Université Clermont Auvergne, Clermont Ferrand, France; 5 Faculty of Veterinary Medicine, Hawassa University, Hawassa, Ethiopia; 6 UCD Centre for Experimental Pathogen Host Research, University College Dublin, Belfield, Dublin, Ireland

**Keywords:** bovine tuberculosis, culture, lesion, *Mycobacterium bovis*, post-mortem inspection, region of difference (RD) typing, whole-genome sequencing

## Abstract

Bovine tuberculosis (bTB), caused by *Mycobacterium bovis* and other members of the *Mycobacterium tuberculosis* complex (MTBC), is a significant concern for livestock and public health in Ethiopia. This study aimed to isolate and genetically characterize the causative agents of bTB in cattle from four abattoirs in the Wolaita zone of Ethiopia. A total of 2,251 cattle were examined post-mortem, and suspect tuberculous lesions were identified in 122 animals. From these animals, 180 tissue samples were collected and processed for bacteriological culture and genetic analysis, including the Loopamp^™^ commercial loop-mediated isothermal amplification kit, PCR targeting RD4 and RD9 loci and whole-genome sequencing (WGS). Bacteriological culture using mycobacteria growth indicator tube and Lowenstein–Jensen media ultimately identified 18 culture-positive samples, with WGS confirming *M. bovis* in lesions from four animals and *M. tuberculosis* in lesions from one animal. The *M. bovis* and * M. tuberculosis* isolates showed genetic similarity to previously identified MTBC lineages in Ethiopia. The presence of *M. tuberculosis* in cattle raises concerns about human-to-animal transmission. Additionally, non-tuberculous mycobacteria were isolated from lesions from multiple animals. Our study genetically characterized bacteria from suspect tuberculous lesions and provides the research community with new genome data for Ethiopian isolates of *M. bovis* and *M. tuberculosis*.

## Data Summary

The authors confirm that all supporting data, code and protocols have been provided within the article or through supplementary data files. Sequence data were submitted to the Sequence Read Archive under NCBI BioProject PRJNA1121228. Individual accession numbers for the sequences in this project are as follows: SRR29318490; SRR29318489; SRR29318488; SRR29318487; SRR29318486; SRR29318485; SRR29318484; SRR29318483; SRR29318482; SRR29318481; SRR29318480; SRR29318479; SRR29318478; SRR29318477. Python Jupyter notebooks and metadata used in this analysis are archived on Zenodo [https://doi.org/10.5281/zenodo.13379235].

## Introduction

Bovine tuberculosis (bTB), caused by members of the *Mycobacterium tuberculosis* complex (MTBC) and in particular *Mycobacterium bovis*, is endemic in Ethiopia, where it poses significant challenges to both livestock and public health. Ethiopia has the largest cattle population in Africa, in excess of 70 million head [1], with over 80% of rural households owning cattle and 57% of livestock-owning urban households having cattle [2]. In terms of public health, zoonotic transmission of *M. bovis* is a major concern. This is particularly the case in areas with high bTB prevalence, where the prevalence of zoonotic Tuberculosis (TB) caused by *M. bovis* is higher than seen elsewhere [3].

The control and management of bTB in Ethiopia face significant challenges due to limited resources and infrastructure. Limited slaughterhouse surveillance and lack of cattle movement controls [4] exacerbate the spread of infection. Exotic breeds, which are often used in dairy farming, are also more susceptible to bTB compared to indigenous breeds [5]. Intensive dairy farms in Addis Ababa and surrounding areas have an overall herd prevalence of 50% [6]. This high prevalence in intensive settings contrasts with the lower overall prevalence of 4.6% reported in a meta-analysis of extensive systems [7]. bTB has negative impacts on productivity traits such as meat production and fertility [8]. The estimated negative economic effects of bTB on the Ethiopian livestock sector are substantial, with a 7.2% reduction in herd productivity and with particular current and future impact on the growing Ethiopian dairying system [9].

In the absence of nationwide bTB surveillance, local abattoir surveys can provide insights into the prevalence of tuberculous lesions so as to assess disease burden in particular regions [10,13]. Post-mortem abattoir inspection aims to detect tuberculous lesions in organs such as the lungs, liver and lymph nodes. Previous abattoir studies have revealed regional variations in bTB prevalence, reflecting differences in local farming practices such as intensive (dairying) or extensive husbandry or factors such as age, sex or breed of animals [10,13].

We set out to identify causative agents of suspect tuberculous lesions in the Wolaita zone in the Southern Ethiopia Region, a zone with over 920,000 cattle [14]. Cattle are a mainstay of the local economy, supplying both ‘*teho bora*’ beef (from cattle specifically raised and fattened for meat production) and ‘*senga*’ beef (often derived from older cattle that are no longer useful for dairy production, traction or other agricultural purposes). Prior to slaughter, cattle are often fattened using the ‘*gatata*’ system, which includes a switch to an enriched diet and enclosure of the animals for ease of husbandry (either indoors or externally). Enclosure of animals in confined spaces would increase the risk of *M. bovis* transmission. A study by Ameni *et al*. [15] using the tuberculin test revealed a disease prevalence of 14.2% in cattle in the Wolaita zone. Furthermore, Hemecho and Tuffa [16], using the comparative tuberculin test, found 96 reactors among 338 tested animals in 38 dairy herds in the Wolaita zone. According to Willeberg *et al*. [17], slaughterhouse surveillance is considered to be a simple, cost-effective and fairly reliable method of detecting animals with bTB with a high level of sensitivity. As the epidemiological situation of bTB in the Wolaita zone is understudied, slaughterhouses therefore represent an important epidemiological starting point for bTB studies in this zone. Therefore, the purpose of our study was to isolate and genetically characterize [using loop-mediated isothermal amplification (LAMP), PCR and genome sequencing] bacteria isolated from suspect tuberculous lesions in tissues collected from slaughtered cattle in the Wolaita Zone.

## Methods

### Study location

This study received ethical approval from the University College Dublin Animal Research Ethics Board (AREC-E-19–19-Gordon) and the Wolaita Sodo University Institutional Review Board (Rf No. ወሶዩ H114532). Four abattoirs in the Wolaita zone were selected for the study based on throughput and accessibility. The Sodo abattoir is the largest local abattoir, processing up to ~46 animals a day. The Areka, Boditti and Humbo abattoirs have lower throughputs of ~20 animals per day. These areas cover diverse agroecological settings and climates, from highland to mid-highland and lowland settings, which in turn allows for a range of agricultural activities, from crop farming to livestock rearing.

### Sampling and sample collection

Collection of samples was carried out from May 2020 to July 2021 by systematic random sampling of slaughtered beef cattle. Veterinary inspection of internal organs (lungs, liver, kidneys, lymph nodes, etc.) was used to detect suspect tuberculous lesions. In total, 2,251 animals were examined: 1,341 animals from Sodo, 349 from Areka, 291 from Boditti and 270 from Humbo abattoirs, respectively. Suspect lesions were aseptically removed, in some cases selecting multiple lesions from the same tissue to maximize the chances of isolating the causative agent. Samples were transported in a chilled cold box to the laboratory (WSU and AHRI), where samples were then processed individually for bacteriological culture and molecular characterization.

### Bacteriological culture

Samples were processed using the method described by Berg *et al*. [18]. Tissue samples were dissected to remove excessive fat or connective tissue and then manually homogenized using a pestle and mortar. Decontamination was performed by adding an equal volume of 4% NaOH for 15 min, and then the suspension was centrifuged at 3,000***g*** for 15 min. Then, the supernatant was removed and the sediment was neutralized by dropwise addition of 2 N HCl, using phenol red as an indicator. The suspension was then used to inoculate two different mycobacteria selective media: premade Lowenstein–Jensen (LJ) media with pyruvate (LJ-P) (Laboratorios Conda, Spain) and mycobacteria growth indicator tube (MGIT, Becton Dickinson) medium supplemented with PANTA antibiotic mix (1:50). The slants were incubated at 37 °C for 8 weeks, while the MGIT tubes were incubated in the BACTEC^™^ MGIT^™^ 960 system (Becton Dickinson) for 8–14 days to report positive results. LJ-P slants were examined on a weekly basis for the presence of mycobacterial colonies; cultures were considered negative if no visible growth was detected after 8 weeks of incubation. Microscopic examination of cultures using the Ziehl–Neelsen (ZN) staining method was performed to select acid-fast (AF) positive isolates. Heat-killed cells were prepared by suspending two loopfuls of bacterial colonies in 500 µl nuclease-free distilled water followed by heat inactivation at 80 °C for 30 mins and then storage at −20 °C. AF-positive cultures were stored as stocks at −80 °C in 20% glycerol (freezing medium).

### DNA extraction

Crude DNA extracts were obtained from suspensions of heat-killed bacterial cells by brief boiling (100 °C for 5 mins), followed by centrifugation to pellet cellular debris and harvesting the supernatant. To purify DNA for genome sequencing, a modification of a previous method was used [19]. Briefly, 0.7 ml of heat-killed *M. bovis* suspension was added to 2 ml screw cap tubes containing 200 µl of 0.1 mm silica beads (Biospec Products Inc). Tubes were placed in a bead beater (MagNALyser, Roche) for three 40 s runs at 6 m s^−1^, and tubes were then centrifuged at 13,000 r.p.m. for 10 min. In brief, 450 µl of supernatant was removed and added to a fresh tube; 45 µl of 3M sodium acetate and 1 ml of ice-cold 96% ethanol were added to each tube, mixed thoroughly and placed at −20 °C for 1 h. The tubes were then centrifuged at 13,000 r.p.m. for 15 mins and the supernatant carefully decanted. DNA pellets were washed in ice-cold 70% ethanol and then air dried for 15 mins. Dried pellets were resuspended in 50 µl Tris-EDTA (TE) buffer and further purified by magnetic bead purification. Briefly, 81 µl of Ampure XP magnetic purification beads (Beckman Coulter) were added to each tube, vortexed for 30 s and incubated for 10 mins at room temperature. The tubes were then placed on a magnetic stand (Invitrogen) for 3 mins, and the supernatant was discarded. Maintaining tubes on the magnetic stand, the pellet was washed twice with 200 µl 80% ethanol, the pellet air dried and beads resuspended in 30 µl of TE. Finally, 28 µl of the supernatant was harvested and stored at 4 (short term) or −20 °C (long term), respectively.

### Loop-mediated isothermal amplification

The Loopamp^™^ LAMP MTBC kit was used (Eiken Chemical Co., Ltd.). This kit amplifies targets in *gyrB* and IS*6110* loci of MTBC. The LAMP protocol was performed according to the manufacturer’s instructions. Briefly, 30 µl of supernatant from crude DNA extractions (see above) was added to each reaction tube. Positive and negative controls were included (provided with the kit). Isothermal amplification was performed at 67 °C for 40 mins followed by 80 °C for 5 mins. Reaction results from test samples were determined as ‘positive’ or ‘negative’ using a hand-held UV transilluminator and with reference to the kit positive and negative controls.

### Polymerase chain reaction

Primers were designed that targeted the RD4 and RD9 loci of the MTBC [20,23]. A combination of three PCR primers was used, two flanking primers and one internal primer, that would result in different size PCR products if the region of difference (RD) was present or absent. The RD9 PCR primers were the same as those previously described [22], while the RD4 primers were designed for this study (Table 1). The general PCR setup conditions were as follows: 4 µl 5× Green GoTaq Flexi Buffer (Promega), 2 µl 25 mM MgCl2 (Promega), 0.5 µl 10 mM dNTPs (Promega), 0.25 μl GoTaq G2 Flexi DNA polymerase (Promega), 1 µl (100 µM) forward primer, 1 µl (100 µM) reverse primer, 1 µl (100 µM) internal primer and 1 µl of template DNA (≥50 ng µl^−1^) and 9.25 µl distilled water, for a total reaction volume of 25 µl. Genomic DNA from *M. tuberculosis* H37Rv and *M. bovis* BCG served as template controls, while molecular grade water was used as the negative template control. The PCR thermal cycles used for RD4 PCRs were 95 °C for 2 mins, followed by 30 cycles of 95 °C for 35 s, 60 °C for 35 s, 72 °C for 35 s and finishing with 72 °C for 5 mins. For RD9, the PCR conditions were 10 mins at 95 °C for enzyme activation, 35 cycles of 1 min at 95 °C for denaturation, 30 s at 61 °C for annealing, 2 mins at 72 °C for extension and a final 10 min extension at 72 °C. The amplified products were run on a 1.5% agarose gel in a Tris-acetate EDTA buffer and visualized with ethidium bromide (RD9) and SYBR Green (RD4). A 100 bp DNA ladder (Invitrogen) was used as a molecular weight standard.

**Table 1.** PCR primers used in this study

Primer	Sequence (5′−3′)	Product size
RD4_FlankF	CTCGTCGAAGGCCACTAAAG	RD4 present=334 bp RD4 deleted=566 bp
RD4_FlankR	AAGGCGAACAGATTCAGCAT	
RD4_InternalF	ACACGCTGGCGAAGTATAGC	
RD9flankF	GTGTAGGTCAGCCCCATCC	RD9 present=396 bp RD9 deleted=575 bp
RD9intR	CTGGACCTCGATGACCACTC	
RD9flankR	GCCCAACAGCTCGACATC	

### Whole-genome sequencing and computational analyses

Illumina whole-genome sequencing (WGS) was performed commercially by Novogene (UK). Libraries (350 bp) were constructed and sequenced on the NovaSeq X Plus platform to generate 150 bp paired-end reads (Q30≥85%). Kraken was used for initial classification of sequence reads into broad taxonomic categories. For read alignment and variant calling of raw sequence data, SNiPgenie [24] was used (https://github.com/dmnfarrell/snipgenie). Reads were aligned to the *M. bovis* AF2122/97 reference genome for *M. bovis* strains and to *M. tuberculosis* H37Rv for the *M. tuberculosis* strains. In both cases, known repeat regions were masked. SNP variants were called, and the resulting core SNP alignment was used to construct a maximum-likelihood tree with RAxML [25] using the GTR-CAT substitution model and 200 bootstrap replicates. Spoligotypes were derived from the raw sequence data using custom Python code that is available within SNiPgenie. MTBC lineages were identified using the web version of TB-profiler [26]. *M. bovis* samples were screened for the presence or absence of the RDAf2 deletion by blasting assembled reads to the sequence of the RDAf2 locus [27]. Species identification for non-MTBC samples was achieved using a custom Python function also available in SNiPgenie. Briefly, the reads were *de novo* assembled using SPAdes [28], and then a local blast to the bacterial 16S NCBI sequences was performed. The results were sorted by percent identity and coverage, and species contributing to the majority of sequence reads were identified. Raw sequence data for representative Ethiopian *M. bovis* strains used in the comparative analyses were downloaded from the Sequence Read Archive (SRA) from BioProject PRJEB32192, with linked metadata for these samples obtained from the study by Almaw and colleagues [29]. Phylogenetic trees were drawn with the ggtree R package [30]. Python Jupyter notebooks and metadata used in this analysis are archived on Zenodo [https://doi.org/10.5281/zenodo.13379235]. Raw sequence data were submitted to the SRA and can be found under NCBI BioProject PRJNA1121228. Lineages for all samples were calculated with TB-profiler. Publicly available genome sequences were downloaded from SRA [31,32].

## Results

### Sample collection

Of the 2,251 animals examined at the four abattoirs, 122 presented suspect tuberculous lesions. All of these animals were male and 5–8 years of age. From these carcasses, a total of 180 tissues featuring lesions were selected, mainly lymph nodes, lungs and livers. These samples were transported back to the laboratory for bacteriological culture.

### Bacteriological culture

In our initial analysis, 180 lesion samples were processed and set up for bacteriological culture on LJ-P slopes. In total, 41 of these samples showed growth suggestive of mycobacteria, of which 32 were AF following ZN staining. Analyses of these samples with both Loopamp^™^ LAMP assay, as a simple colony-screening approach, showed that 24 of these samples were positive for MTBC DNA (visual examination of fluorescence to call a positive vs. the positive and negative controls); however, RD9 PCR analysis of these same samples gave conflicting results, with many negative samples. Given we were concerned that contaminants might have caused the conflicting LAMP and PCR results, we therefore re-cultured the 32 AF-positive lesion samples using both a BD BACTEC^™^ MGIT^™^ 960 system (plus PANTA antibiotic mix) as well as LJ slopes. In this second round of culture using both BACTEC and LJ slopes, 18 of these 32 samples showed positive growth. We focused our subsequent genetic analysis on these 18 culture samples (Table 2).

**Table 2.** Description of isolates and their identification

Animal	Sample	Tissue	RD9 PCR	RD4 PCR	WGS*	Spoligotype	MTBC lineage
**s858**	SH019	Liver	RD9−	RD4−	*M. bovis*	SB1176	Af2
**s858**	SH015	Liver	RD9−	RD4−	*M. bovis*	SB1176	Af2
**s858**	SH016	Liver	RD9−	RD4−	*M. bovis*	SB1176	Af2
**s858**	SH017	Liver	RD9−	NP	*M. peregrinum*	na	na
**s968**	SH20	Liver	RD9−	RD4−	*M. peregrinum*	na	na
**s968**	SH21	Liver	RD9−	RD4−	*Streptomyces*	na	na
**b908**	SH023	Lung	RD9−	RD4−	*M. bovis*	SB1176	Af2
**b908**	SH025	Lung	RD9−	RD4−	*M. bovis*	SB1176	Af2
**a389**	SH024	Lung	RD9+	RD4+	*M. tuberculosis*	L4	L4
**a389**	SH013	Lung	RD9+	RD4+	*M. tuberculosis*	L4	L4
**s103**	SH026	Lung	RD9−	RD4−	*M. bovis*	SB1176	Af2
**h124**	SH005	Lung	NP^†^	NP	*M. nonchromogenicus*	na	na
**s802**	SH010	Liver	NP	NP	*M. nonchromogenicus*	na	na
**a598**	SH014	Liver	NP	NP	*M. peregrinum*	na	na
**h448**	SH018	Lung	RD9−	RD4−	*M. bovis*	Unknown	Af2
**s219**	A004_181	Lung	NP	nd	*N. farcinica*	na	na
**s219**	A004B_182	Lung	nd	nd	*N. farcinica*	na	na
**s219**	S219_183	Lung	nd	nd	*N. farcinica*	na	na

NP,No Product; nd,Not Done; na,Not Applicable. NP†, faint RD9+band present.

*WGS for all samples is available via NCBI BioProject PRJNA1121228 except samples SH20, SH21, A004_181 and A004B_182.

### RD9 and RD4 PCR

**Fig. 1.**
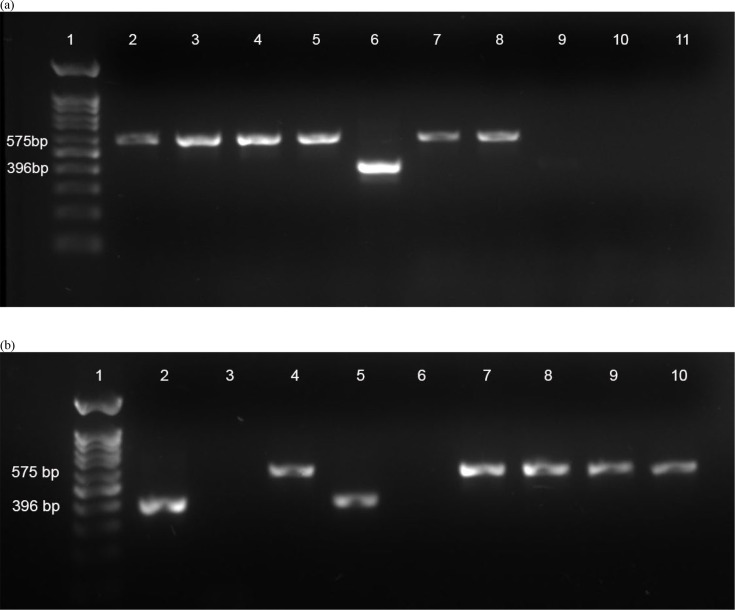
RD9 PCR results. Samples were tested by PCR for the presence or absence of the RD9 locus. Samples with RD9 present (i.e. *M. tuberculosis*) gave a 396 bp product, while samples that had RD9 deleted (i.e. *M. bovis*) gave a 575 bp product. (a) Lanes: 1, 100 bp molecular weight marker; 2, SH19; 3, SH20; 4, SH21; 5, SH23; 6, SH24; 7, SH25; 8, SH26; 9, SH05; 10, SH10; 11, SH14. (b) Lanes: 1, 100 bp molecular weight marker; 2, *M. tuberculosis* H37Rv control; 3, distilled water (no template) control; 4, *M. bovis* BCG control; 5, SH13; 6, A004_181; 7, SH15; 8, SH16; 9, SH17; 10, SH18.

Analysis of the presence or absence of the genomic loci RD9 and RD4 in MTBC allows identification of MTBC species, with * M. bovis* for example having both the RD4 and RD9 loci deleted from its genome (RD4− RD9−), while *M. tuberculosis* has both of these loci present (RD4+ RD9+). PCR analysis of the 18 culture-positive lesion samples revealed that 7 were positive for both RD4− and RD9−, indicative of *M. bovis*, while 2 were positive for both RD4 and RD9, indicative of *M. tuberculosis* (Table 2 and Figs1  2). One sample (SH017) showed RD9 to be deleted, but the RD4 PCR did not give any product. Of the remaining eight samples, none gave products with either RD4 or RD9 PCRs, suggesting that they were not MTBC species. In light of our initial difficulty with reconciling LAMP and PCR results and to ensure definitive identification of the isolates, we therefore moved to WGS of all 18 samples.

**Fig. 2.**
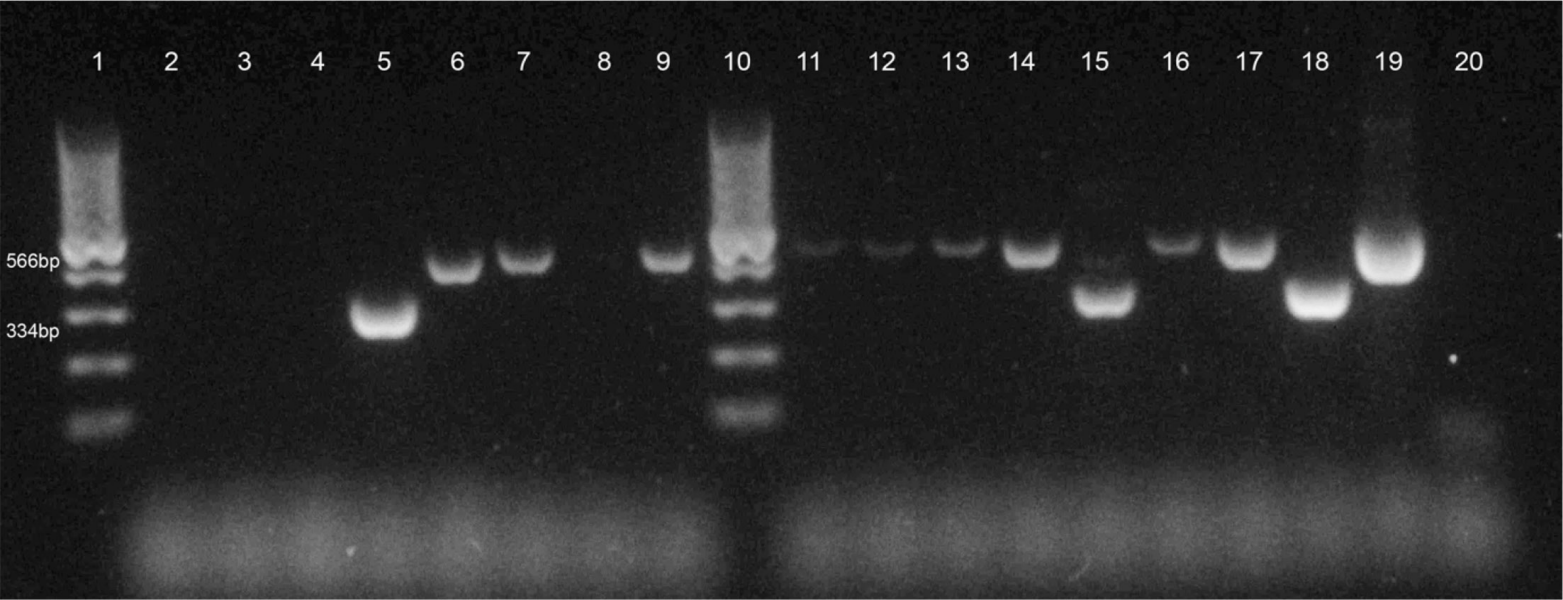
RD4 PCR results. Samples were tested with PCR targeting the RD4 locus. If RD4 was present (i.e. *M. tuberculosis*), a product of 334 bp would be produced, while a product of 566 bp indicated RD4 was deleted (i.e. *M. bovis*). Lanes: 1, 100 bp molecular weight ladder; 2, SH05; 3, SH10; 4, SH14; 5, SH13; 6, SH15; 7, SH16; 8, SH17; 9, SH18; 10, 100 bp ladder; 11, SH19; 12, SH20; 13, SH21; 14, SH23; 15, SH24; 16, SH25; 17, SH26; 18, *M. tuberculosis* H37Rv control; 19, *M. bovis* BCG control; 20, distilled water (no template) control.

### Whole-genome sequencing

**Fig. 3.**
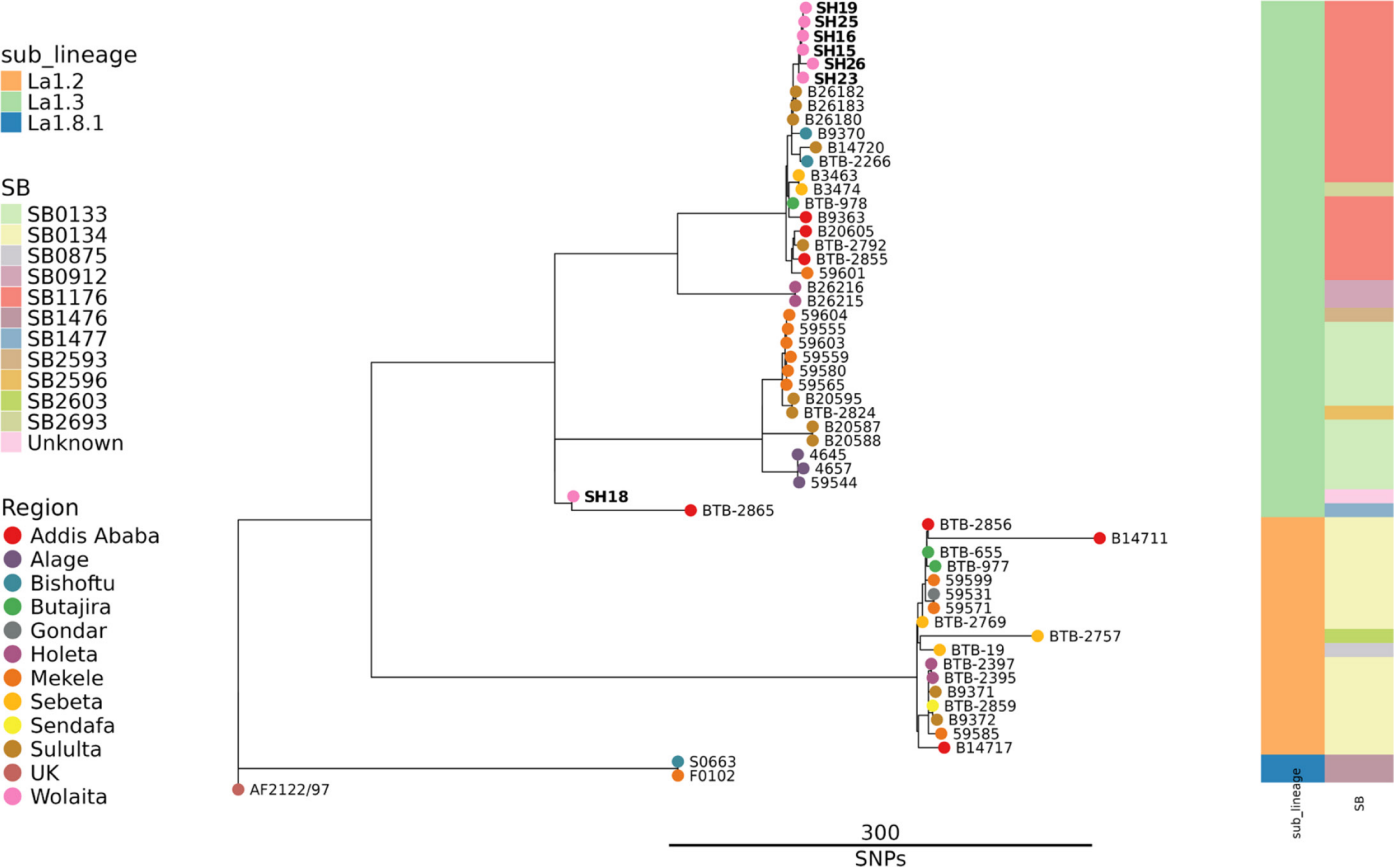
Phylogeny of *M. bovis* strains in this study and representative samples from Almaw *et al.* [29]. Tips are coloured by region of isolation, while to the right are heat maps showing the sub-lineage and spoligotype (SB types, Mbovis.org), respectively. Datasets used for this analysis are available via Zenodo (https://doi.org/10.5281/zenodo.13379235).

All raw sequence data were first checked with Kraken to estimate the make-up of possible species and check for contamination. From this analysis, it was evident that the majority of sequence reads for some isolates were not MTBC species but fell within the Actinobacteria (synonym *Actinomycetota*), with isolates showing genetic similarity to the genera *Mycolicibacterium*, *Mycolicibacter* and *Streptomyces*. Both SH018 and SH019 sequence data contained low to moderate contamination with reads mapping to the bovine genome. Nine samples were determined to contain isolates from the MTBC, and these data were then subject to RD analysis and spoligotyping with tools available in SNiPgenie. Seven isolates were determined to be *M. bovis* and two as *M. tuberculosis*. Subsequent bioinformatic analyses were then tailored if the isolates were *M. bovis* or *M. tuberculosis*. Genome sequence data from the seven *M. bovis* samples were compared to existing *M. bovis* WGS sequence data from Ethiopia [29]. We chose ~5 samples per region from this study to contextualize our isolates from Wolaita within the broader Ethiopian * M. bovis* diversity. Variants were called relative to the *M. bovis* AF2122/97 reference sequence [33]. The resulting phylogeny (Fig. 3) shows that six of the *M. bovis* samples from Wolaita clustered closely with previous *M. bovis* isolates from Ethiopia [29] and all had spoligotype SB1176. Sample SH018 was positioned separately on the tree compared to the other Wolaita isolates but again close to *M. bovis* from Ethiopia [29]. The SH018 spoligotype pattern was novel (1100000101111010111111111110111011111100000) and has not been previously described in the *M. bovis* spoligotype database (https://www.mbovis.org/) now designated as SB2839. SH018 was most closely related to Ethiopian *M. bovis* isolate BTB2865 [29] that has spoligotype SB1477 but which is distinct from the SH018 pattern at three positions (2, 14 and 28).

**Fig. 4.**
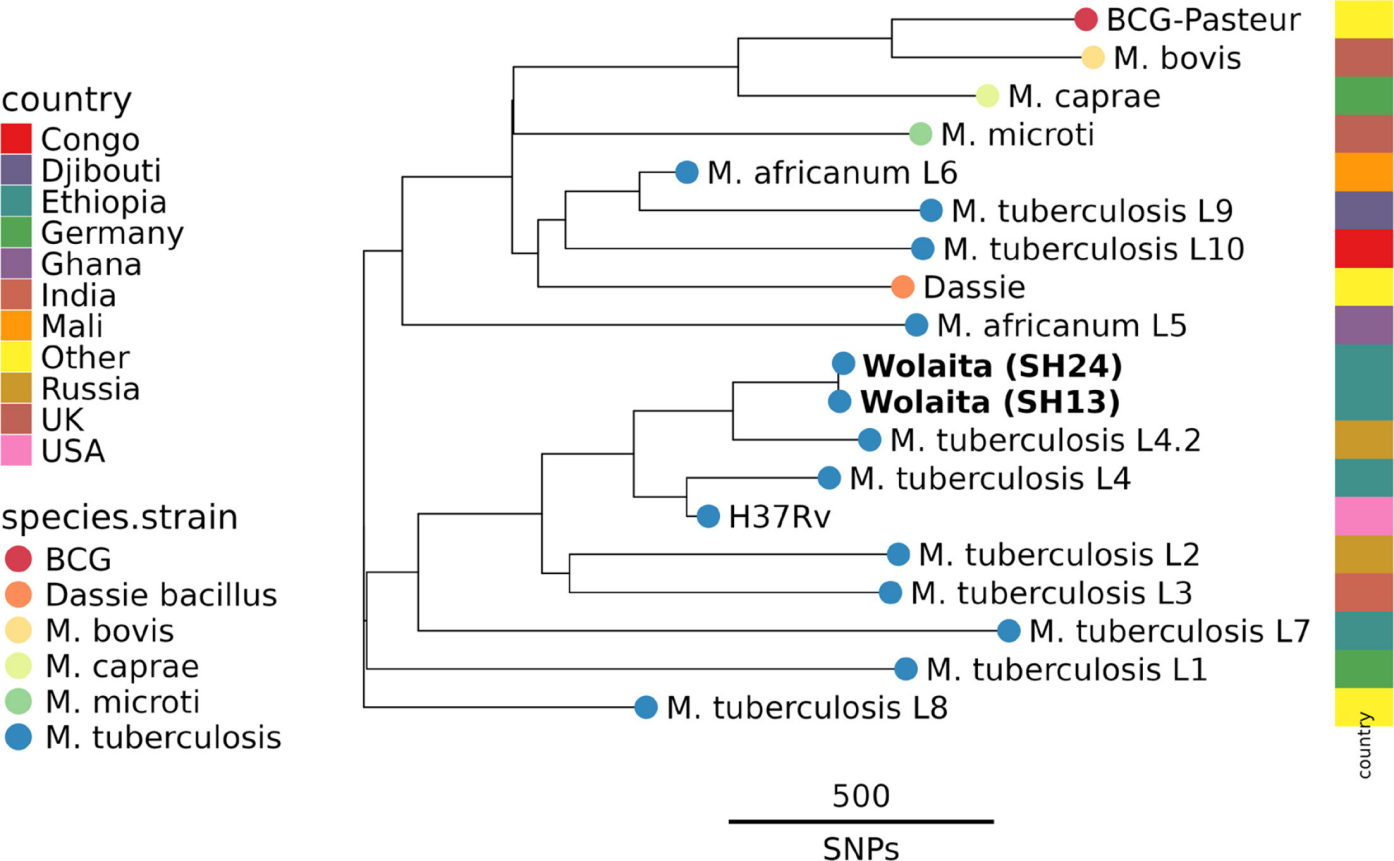
Phylogeny of *M. tuberculosis* strains SH13 and SH24 shown with representative *M. tuberculosis* lineages and MTBC species. Tips are coloured by species with the heat map on the right coloured by country of isolation. The dataset with sample accessions used for this analysis is available via Zenodo (https://doi.org/10.5281/zenodo.13379235).

The WGS data for *M. tuberculosis* isolates SH013 and SH024 (both from animal a389) shared a highly similar SNP profile, with only three SNPs separating them, a result that is consistent with both isolates originating from the same animal. TB-profiler [26] analysis identified both isolates as belonging to *M. tuberculosis* lineage L4.2.2.2 [34]. For phylogenetic analysis, the WGS data for SH013 and SH024 were analysed in combination with WGS data from selected *M. tuberculosis* lineages and MTBC species [32]. From this analysis (Fig. 4), it is also evident that both samples belong to the *M. tuberculosis* lineage 4.

## Discussion

In this work, we set out to identify the causative agents of suspect tuberculous lesions in cattle presenting at four public abattoirs in the Wolaita zone of Ethiopia. We used a combination of bacteriological culture and genetic analyses (LAMP, PCR and WGS) to characterize the isolated bacteria. MTBC complex species were isolated from suspect lesions in the lungs and livers of five animals, four of which were caused by *M. bovis*, while one was due to *M. tuberculosis*. From another six animals, we isolated a range of Actinobacteria, consisting of *Streptomyces*, *Nocardia*, *Mycolicibacterium* and *Mycolicibacter* species.

Previous studies have used similar approaches of culture and genetic analyses to identify bacterial isolates from tuberculous lesions in Ethiopia. Berg *et al*. [18] described a comprehensive slaughterhouse survey of lesions in ~328,000 cattle slaughtered across six different locations in Ethiopia. From these cattle, 1,524 lesions were processed for bacteriological culture and 171 AFB were isolated (~11.2% culture positivity). Genetic analysis of these isolates using RD PCRs (RD4 and RD9) revealed 58 *M*. *bovis* isolates, 8 *M*. *tuberculosis*, 53 NTM and 16 non-*Mycobacterium* isolates. In comparison to the study of Berg *et al*. [18], our lesion collection strategy yielded 32 LJ-P cultures from 180 lesions that were AFB positive (~17.7% positivity). However, given our suspicions of sample contamination, we were concerned about the purity of cultures for subsequent genetic analyses. We therefore re-cultured the 32 lesion samples that had yielded AF-positive cultures on the more selective MGIT plus PANTA antibiotic media as well as standard LJ slopes. This ultimately yielded 18 culture-positive samples from 11 animals, of which 5 were caused by MTBC bacilli. Hence, relative to Berg *et al*., who identified 64 MTBC isolates from 1,524 lesions (~4.3%), our approach yielded 5 MTBC from 180 lesions (~2.7%). While our isolation of MTBC is comparable to that of Berg and colleagues, it could be argued that our lesion collection strategy lacked specificity, with many lesions not caused by MTBC. We had planned to improve diagnostic specificity by initially screening all lesions by histopathology and ZN staining prior to culture, with the aim of only culturing those lesions which contained AF bacilli. However, due to resource limitations, this proved impossible; we were therefore forced to culture all lesions directly, which may explain our reduced yield of *M. bovis* from the lesions processed.

Another aim of our study was to use a LAMP kit to screen culture-positive samples. LAMP technology has advantages over PCR, including the lack of requirement for a thermal cycler and simple visual identification of positive reactions. LAMP has been used by other authors to identify MTBC in clinical samples, including *M. bovis*. For example, Kapalamula *et al*. [35] described the development of a LAMP assay that targets the RD4 deletion. Their assay could detect as few as ten copies of *M. bovis* genomic DNA and gave specific amplification even in the presence of contaminating DNA [35]. Similarly, Zhang *et al*. [36] reported an *M. bovis* LAMP assay that targeted the *mpt83* gene (albeit *mpt83* is not unique to *M. bovis* and is present in other members of the MTBC). A disadvantage with nucleic acid amplification methods is the requirement to use thermosensitive polymerase enzymes that necessitate a cold chain for transport and storage. To overcome this issue, reagents can be dried in a single tube [37], which greatly facilitates transport and storage. This is the case with the Loopamp^™^ MTBC system from Eiken Chemical Co., Ltd., a WHO recommended LAMP kit for detection of MTBC in sputum samples [38]. The Loopamp^™^ MTBC system targets the IS*6110* insertion sequence and *gyrB* gene in the MTBC and hence is not specific for *M. bovis*. We used the Loopamp^™^ system to screen our culture-positive samples for MTBC-positive samples. We found that 24 samples were positive for MTBC by LAMP, but subsequent PCR and WGS found that only 9 of these were identified as *M. bovis* or *M. tuberculosis*. While in our hands we had issues with the specificity of the Loopamp^™^ assay, a more detailed analysis of the technique is warranted. Indeed, in a meta-analysis of molecular detection methods for *M. bovis*, Mabe *et al*. [39] found that the LAMP method of Zhang *et al*. [36] reported the highest sensitivity and specificity of published methods for the detection of *M. bovis* included in the meta-analysis.

To further characterize our culture-positive samples, we used the well-described PCR deletion typing methods that focus on the MTBC RD4 and RD9 regions. Our RD4/RD9 PCRs showed generally consistent results, in that samples that showed RD9 to be deleted also showed the deletion of RD4 (hence *M. bovis*), while RD9 positive samples were also RD4 positive (hence *M. tuberculosis*). However, there were exceptions. Samples SH20 and SH21 from animal s986 gave PCR results indicative of *M. bovis* (RD9−/RD4−); subsequent WGS showed that the majority of reads from these samples in fact mapped to *Mycolicibacterium peregrinum* (SH20) or *Streptomyces* species (SH21). Similarly, sample SH017 (from animal s858) showed RD9 as deleted but no product for RD4; WGS showed the culture was *M. peregrinum*. *Mycolicibacter nonchromogenicus* was also isolated from two samples from two animals (h124 and s802). NTM have previously been isolated from bovine lesions in Ethiopia [18,40, 41], including *Mycobacterium peregrinum* (homotypic synonym *M. peregrinum*) and *Mycobacterium nonchromogenicum* (homotypic synonym *M. nonchromogenicus*). However, we should caution that some of the NTMs isolated in our study may represent contaminants. We suggest this since sample SH017, which was positive for *M. peregrinum*, was from the same animal (s858) as samples SH015, SH016 and SH019, which all gave consistent PCR results (RD9−/RD4−) for *M. bovis*. Furthermore, low levels of sequence reads mapped to *M. bovis* in sample SH20 (while the majority of reads mapped to *M. peregrinum*). A possible explanation for these results is that the lesions originally contained *M. bovis*, but during culture, contaminating overgrowth by more rapidly growing bacteria led to the culture and resulting DNA purified from WGS being dominated by the contaminant. The sensitivity of PCR allowed remaining low levels of *M. bovis* DNA to be amplified, while in WGS, only the dominant contaminant could be identified. Our decision to culture multiple lesions from the same animal was beneficial in this regard, as it provided internal controls and parallel samples in cases of contamination.

Our final step in characterizing our isolates was to use WGS. The most comprehensive genomic analysis of *M. bovis* from Ethiopia published to date is the study of Almaw *et al*. [29]. This latter study reported genome data on 134 *M*. *bovis* isolates from 12 sites across Ethiopia, mainly from dairy cattle but including isolates from slaughterhouse cattle and from human patients. Their analysis revealed distinct spatial distribution of *M. bovis* clonal groups in Ethiopia, with *M. bovis* Af2, a clonal type common in East Africa [27], having a more southerly distribution, while the *M. bovis* EU3 clonal type [42] showed a more northerly distribution. In total, 22 distinct spoligotype patterns were also evident across the sequenced genomes. Our WGS analysis identified * M. bovis* in lesions from four animals (s858, b908, s103 and h448) and *M. tuberculosis* in lesions from one animal (a389) (Table 2). Comparison of our genome sequences to the WGS data of Almaw *et al*. [29] revealed that the Wolaita *M. bovis* were genetically similar to previously described Ethiopian isolates. All our *M. bovis* isolates belonged to the Af2 clonal grouping, in agreement with the spatial distribution of this clonal group described by Almaw *et al*. [29]. Reconstruction of spoligotypes from the sequence reads in the DR region revealed types SB1176 and SB1477 (Table 2), *M. bovis* spoligotypes that have been previously reported as prevalent in Ethiopia [18,29, 43]. *M. tuberculosis* was cultured from two lesions from the same animal (a389), with genome sequencing revealing these isolates to be effectively identical and belonging to the L4 ‘Euro-American’ lineage of *M. tuberculosis* [34], a lineage that is common in Ethiopia [44,45]. *M. tuberculosis* has previously been isolated from cattle in Ethiopia [46], suggesting potential human-to-bovine transmission of infection. This underlines the importance of accurate identification of causative pathogens from suspect tuberculous lesions so as to inform epidemiological understanding of the infection transmission dynamics in the local area.

While the literature indicates that strains of *M. tuberculosis* lineage 4 are attenuated in experimental infection models in cattle [47,48], variations in host immune response or pathogen virulence could lead to productive infections under natural conditions [49,51]. The possibility also exists that co-infection with other pathogens such as flukes could depress immune responses and hence affect susceptibility to *M. tuberculosis* infection [52]. The potential role of management factors, such as close human–cattle interactions in backyard fattening systems, could also elevate the risk of zoonotic *M. tuberculosis* transmission [46,51]. These findings highlight the intricate interactions between *M. tuberculosis* and its hosts, emphasizing the importance of understanding cross-species infections and transmission dynamics of MTBC in livestock and human populations to inform public health and veterinary control strategies for bovine TB [29,53].

In conclusion, we used a combination of bacteriology and genetic analyses to identify mycobacteria associated with suspect tuberculous lesions in the Wolaita zone of Ethiopia. We found that LAMP was a useful screening tool, but that specific identification of isolates required PCRs targeting RD loci, with WGS allowing us to relate the isolates to *M. bovis* and *M. tuberculosis* strains previously described in Ethiopia. Our study highlights the need for combined bacteriological culture and genetic analyses for the accurate identification of mycobacterial pathogens and provides new publicly available genome data for *M. bovis* and * M. tuberculosis* isolates from the Wolaita zone.

## Abbreviations

AF: acid-fast
bTB: bovine tuberculosis
LAMP: loop-mediated isothermal amplification
LJ: Lowenstein–Jensen
LJ-P: LJ media with pyruvate
MGIT: mycobacteria growth indicator tube
MTBC: *Mycobacterium tuberculosis* complex
RD: region of difference
SRA: Sequence Read Archive
TE: Tris-EDTA
WGS: whole-genome sequencing
ZN: Ziehl–Neelsen
